# Supplementary material for: Relationships between External, Wearable Sensor-Based, and Internal Parameters: A Systematic Review
Source: Sensors (Basel). 2023 Jan 11;23(2):827. doi: 10.3390/s23020827 (PMC9864675; doi:10.3390/s23020827)
Supplement: Supplementary file 1 [file sensors-23-00827-s001.zip › sensors-2023568-supplementary.pdf]

sensors
MDPI

Glossar

ACWR acute-to-chronic-workload-ratio
ANN artificial neural network analyses
AU arbitrary units
CI confidence interval
CK creatine kinase
CL confidence level
CMJ countermovement jump
CRP C-reactive protein
ES effect size
GEE Generalized estimating equations
HI high-intensity
HRmax maximal heart rate
HR heart rate
HRV heart rate variability
HS high-speed
HSR high-speed running
LDH Plasma lactate dehydrogenase
LI low intensity
Ln RMSSD natural logarithm of the square root of the mean of the sum of squares of differences between adjacent normal R-R intervals
MAS Maximal aerobic speed
MI moderate intensity
MSR moderate-speed running
NT-proBNP Urinary N-terminal prohormone of brain natriuretic peptide
PA pennation angle
PL PlayerLoad
RHIE repeated high-intensity events
RPE rating of perceived exertion
RSA repeated sprint ability
sIgA salivary secretory immunoglobulin A
T_{core} core temperature
TD total distance
TRIMP training impulse
VO_{2max} maximal oxygen uptake
VT ventilatory threshold
YYIR Yo-Yo intermittent recovery test
$\chi 2$ Wald chi-square.

Table S1. Studies included in this systematic review, the parameters collected and the relationship found between them. Results are listed by sport in alphabetical order.

Sport	Study	Player level (n = number of athletes)	External Parameters (n = number of studies)	Internal Parameters (n = number of studies)	Relationship
**American Football**	[31–36]	University Divison I (n =225, male)	PL (AU) (n = 4)<br>Acceleration/Deceleration ($m \cdot s^{-2}$) (n = 4)<br>Distance in speed zones (m) (n = 2)<br>Impacts (n) (n = 2)<br>Stride variability (n = 1)	INTERNAL LOAD PARAMETERS<br>(session-)RPE (AU) (n = 1)<br><br>EXERCISE-INDUCED RESPONSES<br>Well-being questionnaire (5-point scale) (n = 4)<br>S100beta (pg/ml) (n = 1)<br>Tau concentration (pg/ml) (n = 1)	INTERNAL LOAD PARAMETERS<br>**(session-)RPE and …**<br>… PL:<br>Trivially related using linear mixed models (χ^2 (1) = 137.5, p<0.01, standardized mean difference: 0.01): a 1-unit increase in PL → 0.3% (90% CI: 0.2; 0.3) increase in session-RPE [31]<br><br>EXERCISE-INDUCED RESPONSES<br>**Well-being questionnaire and…**<br>… PL:<br>Trivially related using linear mixed models: 1-unit increase in wellness Z-score → 2.3% (CI: 0.5; 4.2; SMD 0.12) increase in PL [31]<br>Inversely significantly related using multilevel mixed linear regressions: significantly lower wellness scores in players with higher PL values (p<0.05) [35,36]<br>… acceleration/deceleration:<br>Significantly more acceleration and deceleration distance covered the preceding day for players with higher perceived fatigue and for those with higher perceived soreness than for less fatigued and less sore players, respectively (p<0.05) [35]<br>… total distance:<br>Significantly more distance covered the preceding day for players with higher perceived soreness and for those with higher perceived fatigue than for less sore or less fatigued players, respectively (p<0.05) [35]<br>… distance in speed zones:

					Significantly more LI, MI, and HI distance covered the preceding day for players with higher perceived soreness than for less sore players (p<0.05) [35]<br>… stride variability:<br>1-point increase in fatigue score (feeling better) related to 0.508% decrease in between-stride coefficient of multiple determination (meaning increased stride variability) during HSR (≥ 2.75 steps/s for at least 5 seconds) using linear mixed models (95% CI: -0.953, -0.063%, p<0.05) [34]<br>**S100beta levels and …**<br>… head impacts, head peak linear, and peak rotational acceleration:<br>Significantly relationship between number of hits and pre-post practice increases in S100beta levels (F (1, 70) = 11.54, p=0.001) as well as the sum of head peak linear (F (1,70) = 8.78, p = 0.004) and peak rotational acceleration (F (1,70) = 14.05) using mixed effects regression models (p≤0.001) [32]<br>**Tau concentration and …**<br>… head impacts:<br>No relationship found between head impacts and tau concentration using mixed-effect regression models [33]
**Australian Football**	[13,37–45]	Professional (n = 202, male)<br>Elite (n = 118, male)	Distance in speed zones (m) (n = 13)<br>PL (AU) (n = 9)<br>Total/Relative distance (m, m/min) (n = 9)<br>Duration (min) (n = 5)<br>Average speed (m/s)(n = 4)	INTERNAL LOAD PARAMETERS<br>(session-)RPE (AU) (n = 7)<br>Core temperature (C) (n = 1)<br><br>EXERCISE-INDUCED RESPONSES<br>Well-being questionnaire (5-point scale) (n = 3)<br>CMJ (cm) (n = 1)	INTERNAL LOAD PARAMETERS<br>**(session-)RPE and …**<br>… GPS measures (PL (3D & 2D), low- & high-speed running distance, total/relative distance, total/relative high power distance, estimated energy expenditure, metabolic power, equivalent distance):<br>Small within-player correlations between RPE and external parameters for RPE-match exertion (r = 0.14 - 0.28), unclear to small for RPE-breathlessness (r = 0.06 - 0.24) and unclear to moderate for RPE-leg exertion (r = 0.06-0.37) [44]<br>…. distance in speed zones:<br>HSR (>14.4km/h) was predictive of RPE in 3 of 41 players using ANN [13]<br>Large correlation between HSR (>14.4 km/h) and RPE (ρ = 0.69; 95% CI: 0.67; 0.71; p<0.001) [13]

	Acceleration/Deceleration (m·s^{-2}) (n = 3)<br>Energy expenditure (kJ/kg) (n = 2)<br>Metabolic power concept (W/kg) (n = 2)<br>Distance load (distance x mean speed) (m^2/s) (n = 1)<br>Effort zones (n) (n = 1)<br>Equivalent distance (m) (n = 1)<br>Explosive efforts (n = 1)<br>Impacts (n) (n = 1)<br>Match exercise intensity (AU) (n = 1)	CK (U/L) (n = 1)<br><br>INDIVIDUAL CHARACTERISTICS<br>Maximal aerobic speed (m/s) (n = 1)<br>YYIR (m) (n = 1)	Moderate correlation between session-RPE and HSR distance (individual to mean 2km time trial speed) (r = 0.51, 95% CI: 0.42–0.59) [43]<br>… PL :<br>Large correlation between session-RPE and PL and PLslow (r = 0.80-0.86) [43]<br>… total/relative distance:<br>Session distance as the strongest predictor of RPE in 36 of 41 players using predictive models (GEE & ANN) [13]<br>Moderate to large correlation between RPE and relative and total distance (ρ = 0.49; 95% CI: 0.46; 0.52; ρ = 0.77; 95% CI: 0.75; 0.79, respectively; p <0.001) [13]<br>Large correlation between session-RPE and total distance (r = 0.88, 95% CI: 0.85–0.90) [43]<br>Relative distance (m/min) was predictive of RPE in 2 of 41 players using ANN [13]<br>Relative distance as most accurate RPE predictor using random forest models (RMSE ± SD = 0.96 ± 0.08 au) [37]<br>… average speed:<br>Moderate correlation: session-RPE and average speed (r = 0.45, 95% CI: 0.35–0.54) [43]<br>… accelerations/decelerations:<br>Players scoring high on RPE (≥9) covered significantly higher distances in high deceleration (5.9%), medium deceleration (3.2%), and medium acceleration (2.8%) than players with RPE ≤ 8 [41]<br>… distance load:<br>Using a simplified random forest model, RPE could be predicted using total distance, distance between 18-24 km/h, and distance load (RMSE ± SD = 1.09 ± 0.05 au) [37]<br>… metabolic power concept:<br>No differences in metabolic power between players with RPE ≥9 and ≤8 [41]<br>… match exercise intensity (calculated using distance/min and distance at high intensity (>15km/h)/min):

	Very large association: Estimates of match exercise intensity/minute showed very large associations with actual match exercise intensity/minute using session-RPE_{Skills} 0.73 ± 0.14 (r, 90% CI) as input [40]<br>Precision of actual match exercise intensity/min was lowest using session-RPE [40]<br>**Core temperature (T_{core}) and …**<br><u>… distance in speed zones:</u><br>Significant correlations between first-quarter rise in T_{core} and…<br>… HI running velocity (>14.4 km/h) (r = 0.72)<br>… MI velocity (r = 0.68) [95]<br>Significant correlations between second-quarter T_{core} and…<br>… LI activity velocity (r = -0.90)<br>… MI velocity (r = 0.88) [95]<br>Significant correlations between fourth-quarter rise in T_{core} and…<br>… very-HI distance (>14.4 km/h) (r = 0.70)<br>… MI velocity (r = 0.73) [95]<br><br>EXERCISE-INDUCED RESPONSES<br>**Well-being questionnaire and…**<br><u>… PL:</u><br>-1 in wellness Z-score pre-training corresponded to a trivial -4.9 ± 3.1 and -8.6 ± 3.9% reduction in PL/min and PL slow/min, respectively, compared to those without reduced wellness [38]<br><u>… PL & RPE:</u><br>-1 in wellness Z-score pre-training corresponded to a -0.02 ± 0.01 au/min change in PL slow:RPE ratio, compared to those without reduced wellness [38]<br><u>… PL & average speed:</u><br>-1 in wellness Z-score pre-training corresponded to a trivial 0.43 ± 0.38 m/min change in the average speed:RPE ratios, compared to those without reduced wellness [38]<br>**CMJ (as an indirect marker of neuromuscular fatigue) and …**<br><u>… acceleration/deceleration:</u>

					CMJ reductions likely came along with reductions in the vertical acceleration vector (mean +/- 90% CI: -5.8% +/- 6.1%) [45]<br>**CK and…**<br><u>… distance in speed zones</u><br>Sprint distance (>25.2 km/h) as a strong predictor using random forest [39]<br><u>… duration:</u><br>Strong association using generalized estimating equations: Longer game time (χ^2 = 5.77, p<0.05) → higher post-match CK [39]<br><u>… acceleration/deceleration:</u><br>Acceleration/deceleration was a strong predictor using random forest [39]<br><u>… impacts:</u><br>Strong association using GEE: higher number of impacts >3g (χ^2 = 8.14, p<0.005) → higher post-match CK [39]<br><br>INDIVIDUAL CHARACTERISTICS<br>**YYIR (as a marker of intermittent endurance) and** …<br><u>…PL:</u><br>For non-fatigued players (as indicated by a CMJ), practically important correlations existed between the vertical component of PL (r = -0.5 ± 0.3 (CI)), and the medio-lateral component of PL (r = 0.69 ± 0.21 (CI)) and YYIR2 performance [45]<br>**MAS (as a marker of aerobic endurance) and…**<br><u>… distances in speed zones:</u><br>Moderate positive effects of MAS on HSR distance (>20 km/h) (ES: 0.47) [42]<br><u>… total/relative distance:</u><br>Large positive effects of MAS on relative distance (ES: 0.55) [42]
**Basketball**	[46–49]	Elite (n = 12, male)<br>Professional (n = 26, male)	PL (AU) (n = 4)<br>Acceleration/Deceleration (m·s^{-2}) (n = 4)	INTERNAL LOAD PARAMETERS<br>(session-)RPE (AU) (n = 3)	INTERNAL LOAD PARAMETERS<br>**(session-)RPE and …**<br><u>… PL:</u><br>Significant moderate relationships between session-RPE and PL (r = 0.49, 95% CI: 0.23; 0.69, p<0.001) [47]

	Semiprofessional (n = 8, male)<br>University (n = 5, female)	Jumps (n) (n = 2)<br>IMA™ (AU) (n = 1)	HR-based indices (n = 1)<br><br>EXERCISE-INDUCED RESPONSES<br>Tensiomyography (ms, mm) (n = 1)	Very strong correlations between session-RPE and PL variables (PL, PL in the anterior/posterior plane, PL in the lateral plane, PL in the vertical plane) (r > 0.8, p<0.01) [48]<br><u>… acceleration/deceleration (in zones):</u><br>Significant correlations between RPE as well as session-RPE and total forward acceleration (r = 0.48-0.61 for RPE, r = 0.6-0.71 for session-RPE), forward acceleration >3.5 m/s^2 (r = 0.31-0.43 for RPE, r = 0.46-0.58 for session-RPE), total deceleration (r = 0.49-0.72 for RPE, r = 0.68-0.81 for session-RPE), deceleration <-3.5 m/s^2 (r = 0.26-0.56 for RPE, r = 0.45-0.67 for session-RPE) (p<0.01) [48,49]<br>Significant large correlations between RPE as well as session-RPE and total rightward/leftward lateral acceleration movements (r = 0.76-0.78 for RPE, r = 0.75-0.78 for session-RPE), movements in a rightward/leftward lateral vector >±3.5 m/s^2 (r = 0.38-0.41 for RPE, r = 0.48-0.58 for session-RPE) (p<0.01) [48,49]<br><u>… jumps:</u><br>Significant correlations between RPE as well as session-RPE and jump variables: total jumps (r = 0.12-0.40 for RPE, r = 0.32-0.45 for session-RPE), jumps above 0.4 m (r = 0.06-0.58 for RPE, r = 0.31-66 for session-RPE) p<0.01 [48,49]<br>**HR-based indices and …**<br><u>… PL:</u><br>TRIMP and PL:<br>Large correlation between Edwards' TRIMP and PL (r = 0.61, 95% CI = 0.38-0.77, p<0.001) [47]<br>Moderate correlation between Banister's TRIMP and PL (r = 0.38, 95% CI = 0.09-0.61, p<0.05) [47]<br><br>EXERCISE-INDUCED RESPONSES<br>**Tensiomyography and …**<br><u>… acceleration/deceleration (in zones):</u><br>Significant positive correlations between aggregated contraction time of rectus femoris, bicep femoris and adductor longus and anteroposterior acceleration and deceleration (r = 0.41; p<0.01) [46]

					… jumps:<br>Significant positive correlations between aggregated contraction time of rectus femoris, bicep femoris and adductor longus and vertical jumps (r = 0.38; p<0.01) [46]<br>… Inertial movement analysis (IMA™):<br>Significant positive correlations between aggregated contraction time of rectus femoris, bicep femoris and adductor longus and total IMA™ (r = 0.43; p<0.01) [46]
**Field Hockey**	[50]	Elite (n = 12, male)	Acceleration/Deceleration ($m \cdot s^{-2}$) (n = 1)<br>Distances in speed zones (m) (n = 1)<br>Total/relative distance (m, m/min) (n = 1)	EXERCISE-INDUCED RESPONSES<br>Well-being questionnaire (5-point scale) (n = 1)	EXERCISE-INDUCED RESPONSES<br>**Well-being questionnaire and …**<br>… acceleration/deceleration (in speed zones):<br>Very large to nearly perfect associations between Δ in pre-game wellness and efforts/min/RPE of Δ in HI accelerations (≥2 m/s^2) (r = −0.87; CI: −1.00; −0.66) and Δ in HI decelerations (≤-2 m/s^2) (r = -0.90 CI -0.99; -0.74) (p<0.05) [50]<br>… distance in speed zones:<br>Nearly perfect correlations between Δ in pre-game wellness and Δ in m/min/RPE of HI running distance (>15 km/h) (r = −0.95; CI: −1.00; −0.83), and low-intensity activity (<15 km/h) (r = −0.94; CI: −1.00; −0.81) (p<0.05) [50]<br>… total/relative distance:<br>Very large correlations between Δ in pre-game wellness and Δ in m/min/RPE of total distance (r = −0.95; CI: −1.00; −0.82, p<0.05) [50]
**Rugby Sevens**	[51,52]	Elite (n = 24, 12 female, 12 male)<br>Amateur (n = 10, female)	Total/relative distance (m, m/min) (n = 2)<br>Distance in speed zones (m) (n = 2)<br>Impacts (n) (n = 1)	EXERCISE-INDUCED RESPONSES<br>CK (U/L) (n =1)<br>Bicarbonate concentration (mmol/L) (n = 1)<br>Lactate concentration (mmol/L) (n = 1)<br>pH (n=1)	EXERCISE-INDUCED RESPONSES<br>**pH and …**<br>… distance in speed zones:<br>Small negative correlation between relative distance covered at high intensity (> 100% of MAS) and very HI (100% MAS+30%ASR) and pH (r =-0.44 and -0.26, respectively, p<0.01) [52]<br>… total/relative distance<br>No relationship between total or relative distance and pH concentration [52]<br>**Lactate concentration and …**<br>… distance in speed zones:

					Small to moderate correlation between relative distance covered at HI (> 100% of MAS) and very HI (100% MAS+30%ASR) and lactate concentration (r = 0.36 and 0.27, respectively, p<0.01) [52]<br><u>… total/relative distance</u><br>Moderate correlation between TD and very HI distance (100% MAS+30%ASR) during the 1-min peak activity in the last 3 min of play and lactate concentration (r = 0.39; p<0.01) [52]<br>No relationship between total or relative distance and lactate concentration [52]<br>**Bicarbonate concentration and …**<br><u>… distance in speed zones:</u><br>Small to moderate negative correlations between HI (> 100% of MAS) and very HI distance (100% MAS+30%ASR) and bicarbonate concentration (r = -0.42 and -0.28, respectively, p<0.01) [52]<br><u>… total/relative distance</u><br>No relationship between total or relative distance and pH concentration [52]<br>**CK and …**<br><u>… distance in speed zones:</u><br>Large correlation between HSR (>18km/h) and changes in CK (r = 0.66-0.91) [51]<br><u>… impacts:</u><br>Large correlation between number of impacts >10g and changes in CK (r = 0.66-0.91) [51]
**Rugby League**	[53–56]	Professional (n = 46, male)<br>Elite (n = 45, male)	Distance in speed zones (m) (n = 3)<br>Impacts (n) (n = 3)<br>Acceleration/Deceleration ($m \cdot s^{-2}$) (n = 2)	INTERNAL LOAD PARAMETERS<br>(session-)RPE (AU) (n = 2)<br><br>EXERCISE-INDUCED RESPONSES	INTERNAL LOAD PARAMETERS<br>**(session-)RPE and …**<br><u>… external load parameters:</u><br>62.4% of the adjusted variance in session-RPE could be explained by distance, impacts, PL, and TRIMP (y = 37.21 + 0.93 distance – 0.39 impacts + 0.18 PL + 0.03 TRIMP) and 35.2% of the adjusted variance in session-RPE could be explained by % peak HR, impacts/min, relative distance, and PL/min (y = -0.01 + 0.37% HRpeak + 0.10 impacts/min + 0.17 m/min + 0.09 PL/min) using stepwise multiple-regression analysis [53]

	Total/Relative distance (m, m/min) (n = 2)<br>Duration (min) (n = 1)<br>PL (AU) (n = 1)<br>RHIE (n) (n = 1)	Well-being questionnaire (5-point scale) (n = 1)<br>CK (U/L) (n = 2)<br>Salivary cortisol (nmol/L) (n = 1)<br>Repeated plyometric push-ups (n) (n = 1)<br>Sleep (h) (n = 1)<br><br>ADAPTATION PARAMETERS<br>Sleep (h) (n = 1)	EXERCISE-INDUCED RESPONSES<br>**Well-being questionnaire and …**<br><u>… impacts:</u><br>Significant correlations between perceived muscle soreness and total impacts (r = -0.68, CI: -0.87; -0.10, p<0.05) [56]<br><u>… RHIE:</u><br>Significant correlations between perceived muscle soreness and RHIE (r = -0.66, CI: -.89; .21, p<0.05) [56]<br>**CK and …**<br><u>… distance in speed zones:</u><br>Large correlations between HI distance (>18 km/h) and increased CK concentration 12h post-match (r = 0.76, CI: .51; .91, p<0.05) [56]<br><u>… impacts</u><br>Large correlation between the number of impacts between 8.1-10.0 g and >10.1g during matches and CK 30 min, 12 h, 24 h, 48 h, and 72 h post-match (r = 0.62-0.67, p<0.05) [55,56]<br><u>… total/relative distance:</u><br>Large correlation between total distance and increased CK concentration post-match (r = 0.86, CI: .70-.95, p<0.05) [56]<br><u>… duration:</u><br>Duration was associated with increased CK concentration 12h post-match (r = 0.90, CI: .77-.96, p<0.05) [56]<br><u>… RHIE:</u><br>Large correlations between total amount of RHIE and CK concentration 12h post-match (r = 0.73, CI: .51-.87, p<0.05) [56]<br>**Salivary cortisol and …**<br><u>… impacts:</u><br>No significant relationship between salivary cortisol and number of tackles, number of hit-ups, or number of impacts >7.0g during 120 h post-match [55]<br>**Repeated plyometric push-ups (as a marker of neuromuscular functioning) and …**<br><u>… impacts:</u>

					Total collisions were associated with large decrements in upper-body neuromuscular performance (r = -0.48, CI: -.74 to .02, p<0.05) [56]<br><u>… RHIE:</u><br>RHIE were associated with large decrements in upper-body neuromuscular performance (r = -0.49, CI: -.77; .05, p<0.05) [56]<br>**Sleep and …**<br><u>… acceleration/deceleration:</u><br>Higher daily acceleration/deceleration load had small correlations with an increase in sleep efficiency (r = 0.15; ±0.09) and sleep duration (r = 0.12; ±0.09) [54]<br><br>ADAPTATION PARAMETERS<br>**Sleep and …**<br>Greater 3-day weighted and increased 7-day exponentially moving average of acceleration/deceleration load had small correlations with increased sleep efficiency (ES = 0.14-0.15; ±0.09) and an earlier bedtime (r = 0.14-0.15; ±0.09) [54]
**Rugby Union**	[57,58]	Professional (n = 51, male)	Distance in speed zones (m) (n = 2)<br>Impacts (n) (n = 2)<br>PL (AU) (n = 1)<br>Total/Relative distance (m, m/min)(n = 1)	EXERCISE-INDUCED RESPONSES<br>CK (U/L) (n = 1)<br>Urinary N-terminal prohormone of brain natriuretic peptide (pg/mL) (n = 1)	EXERCISE-INDUCED RESPONSES<br>**CK and …**<br><u>… distance in speed zones</u><br>Moderate significant effect-size correlations between HSR (>18km/h) (r = 0.437, p<0.05) and sprinting (>20km/h) (r = 0.41-0.42, p<0.05) distances and ΔCK at 16h (for sprinting only) and 40h post-match in backs only. No significant correlations in forwards [57]<br><u>… impacts:</u><br>Moderate effect-size correlations between total impacts and ΔCK at 16 post-match in backs only (r = 0.638, p<0.01). In forwards, effects were small to moderate and non-significant except for %ΔCK 16h post-match [57]<br>**Urinary N-terminal prohormone of brain natriuretic peptide (NT-proBNP) and …**<br><u>… external load parameters</u> (impacts, total distance, distance in speed zones, PL):

					Significant increase in urinary NT-proBNP during game one did not correlate with the external load parameters [58]
**Soccer**	[59,60,69–78,61,79–88,62,89–93,63–68]	Professional (n = 311, male)<br>Elite (n = 236, male)<br>Semi-professional (n = 61, male)<br>University (n = 114, 79 male, 35 female)	Distance in speed zones (m) (n = 31)<br>Total/Relative distance (m, m/min) (n = 30)<br>PL (AU) (n = 15)<br>Acceleration /Deceleration ($m \cdot s^{-2}$) (n = 13)<br>Duration (min) (n = 12)<br>Impacts (n) (n = 5)<br>Average Speed (m/s) (n = 4)<br>Dynamic stress load (AU) (n = 4)<br>Metabolic power concept (W/kg) (n = 4)<br>Maximal velocity (m/s) (n = 3)<br>Effindex (AU) (n = 2)<br>RHIE (n) (n = 2)<br>Body load (AU) (n = 1)<br>Energy expenditure (kJ/kg) (n = 2)	INTERNAL LOAD PARAMETERS<br>HR-based indices (n = 17)<br>(session-)RPE (AU) (n = 16)<br>Effindex (AU) (n = 2)<br><br>EXERCISE-INDUCED RESPONSES<br>Well-being questionnaire (5-point scale) (n = 8)<br>CMJ (cm) (n = 6)<br>CK (U/L) (n = 5)<br>Immunoglobulin (µg/mL) (n = 3)<br>C-reactive protein (mg/L) (n = 1)<br>HR-based indices (n = 1)<br>Myoglobin concentration (ng/ml) (n = 1)<br>Plasma lactate dehydrogenase (U/L) (n = 1)<br>Body mass measures (kg) (n = 1)	INTERNAL LOAD PARAMETERS<br>**HR-based indices and …**<br><u>… distances in speed zones:</u><br>HS-based parameters (>14.4 km/h) was not a strong predictor of predicted HR, explaining <30% of the total variance using multiple stepwise bidirectional regression analysis [65]<br>Moderate to large correlations between time spent in the low-intensity (LI) velocity zone (<34% of max. velocity) and the LI HR zone (below ventilatory threshold (VT)) (r = 0.46) and between absolute and relative time spent in the moderate intensity velocity zone (34-61% of max. velocity) and the MI HR zone (between VT and respiratory compensation point (RCP)) (r = 0.25-0.57) (p<0.01) (Sparks et al., 2017)<br>No significant correlations between HI velocity zones (>61% of the max. velocity) and HI HR zone (above RCP) (p > 0.01) [80]<br>Small adjusted correlations between HSR distance (>14.4km/h) and the percentage of time spent above 80% of individual HR_{max} (r = 0.18, 95% CI: .06-.29; p<0.05) [79]<br>Small significant correlations between changes in submaximal HR and in 4-day total HSR distance (>14.4km/h) (r = 0.28; p<0.05) [84]<br><u>TRIMP and distances in speed zones:</u><br>Large, significant correlations between low-speed distance (<14.4 km/h) and (Edwards' & Banister's) TRIMP (r = 0.72-0.77; CI: .61-.84, p<0.01) [59]<br>Large to moderate correlations between HSR (>14.4 km/h) and very HSR (>19.8 km/h) distances and (Edwards' & Banister's) TRIMP (r = 0.58-0.62, 0.40-0.41, respectively) (p<0.01) [59]<br>Moderate to trivial significant correlations between frequency of efforts at HS (≥18 km/h), HS distance, and frequency of efforts at sprint speed (≥21 km/h) and Edwards' TRIMP (r = 0.37, 0.25, and 0.18, respectively, p<0.01) [60]<br><u>… total/relative distance:</u>

	Equivalent distance (m) (n = 1)<br>Explosive distance (m) (n = 1)<br>Impulse Load (Ns) (n = 1)<br>Force load (AU) (n = 1)<br>Mechanical work (AU) (n = 1)<br>Training load score by Polar (AU) (n = 1)<br>Total accelerometer load (n = 1)<br>Total forces (n = 1)<br>Velocity load (n = 1)<br>Work:rest ratio (n = 1)	ADAPTATION PARAMETERS<br>HR-based indices (n = 2)<br>Body mass measures (kg) (n = 2)<br>Strength test (Nm) (n = 1)<br>VO_{2max} (ml/kg/min) (n = 1)<br>30-15 intermittent fitness test (m) (n = 1)<br><br>INDIVIDUAL CHARACTERISTICS<br>VO_{2max} (ml/kg/min) (n = 1)<br>YYIR (m) (n = 1)<br>Repeated sprint ability (m) (n = 1)<br>Body mass measures (kg) (n = 1)<br>Muscle characteristics (cm) (n = 1)<br>Sprint test (s) (n = 1)	Total distance was not a strong predictor of predicted HR, explaining <30% of the total variance using multiple stepwise bidirectional regression analysis [65]<br><u>TRIMP and total/relative distance:</u><br>Large significant correlations between Edwards' TRIMP and total distance ($r = 0.72$, $p<0.01$) [60]<br><u>… PL:</u><br>Significant correlations between PL and LI and MI absolute and relative HR zones ($r = 0.24$-0.61; $p<0.01$) [80]<br>No significant correlation between HI HR zone and PL ($p>0.01$) [80]<br><u>TRIMP and PL:</u><br>Large, significant correlations between PL and (Edwards' & Banister's) TRIMP ($r = 0.70$-0.80; CI: .62-.86, $p<0.01$) [59,60]<br>Large to very large within-individual correlations between PL and Edwards' and Banister's TRIMP in 10 out of 11 players ($r = 0.56$-0.89) [72]<br><u>… acceleration/deceleration:</u><br>Small adjusted correlations between number of accelerations >2.5m/s²/min and % time spent above 80% of individual HR_{max} using general linear models ($r = 0.3$; 95% CI: .22-.43; $p<0.001$) [79]<br><u>TRIMP and acceleration/deceleration:</u><br>Moderate adjusted correlations between number of accelerations >2.5 m/s^2 and Banister's TRIMP using general linear models ($r = 0.49$; 95% CI: .39-.58; $p<0.001$) [79]<br><u>… RHIE:</u><br>Small adjusted correlations between number of RHIE and Banister's TRIMP using general linear models ($r = 0.3$; 95% CI: .18-.4; $p<0.001$) [79]<br>Small adjusted correlations between RHIE/min and the % time spent above 80% of individual HR_{max} ($r = 0.22$; 95% CI: .1-33; $p<0.001$) [79]<br><u>… force load:</u><br>Force load[1] as a strong predictor of predicted HR using multiple stepwise regression analysis explaining 31 ± 17% of the total variance [65]<br><u>… mechanical work:</u>

[1] the sum of the estimated ground reaction forces during foot impacts [65]

	Mechanical work[2] as a strong predictor of predicted HR using multiple stepwise regression analysis explaining 24 ± 8% of the total variance [65]<br>**(session-)RPE and …**<br><u>… distance in speed zones:</u><br>Large, significant correlations between LS distance (<14.4 km/h) and session-RPE (r = 0.80; CI: .71-.86, p<0.01) [59]<br>Large to moderate significant correlations between HSR (>14.4 km/h) and very HSR (>19.8 km/h) distances and session-RPE (r = 0.65; CI: .51-.75, r = 0.43; CI: .26-.58, respectively, p<0.01) [59]<br>Large correlations between frequency of efforts at high speed (≥18 km/h) and session-RPE (r = 0.64, p<0.01) [60]<br>Small partial correlations between HSR (>14.4 km/h) and session-RPE (r = 0.11) and between HSR/min and RPE (r = 0.14) (p<0.001) [92]<br>Large to very large within-individual correlation between HSR and session-RPE (r = 0.61) and small between HSR/min and RPE (r = 0.26) (p<0.001) [92]<br>Number of sprints (> 25 km/h) among the strongest predictors of session-RPE using Gradient Boosting Machines [61]<br>Distances covered between 12-15, 15-20, and 20-25 km/h as main contributors to RPE using least absolute shrinkage and selection operator (importance score: 0.487, 0.507, 0.428, respectively) [64]<br>Moderate significant correlations between RPE/session-RPE and MSR (14.4-19.8 km/h) and HSR (19.9-25.1 km/h) when using not individualized speed thresholds (r = 0.53-0.59; p < 0.05) → higher correlations with individualized speed thresholds (r = 0.58-0.67; p < 0.05) [73]<br>Moderate significant correlations between RPE/session-RPE and sprinting distance only when using individualized speed thresholds (r = 0.53-0.55; CI: .02-0.83; p<0.05) [73]<br>Large significant correlations between session-RPE and the sum of distance covered between 14.4 - ≥25.2 km/h, irrespective if speed thresholds were individualized or not (r = 0.58-0.68; p < 0.05) [73]

[2] calculated using >2.ms^{-2} accelerations, decelerations and COD events [65]

	No significant correlations between RPE or session-RPE and % distances in speed zones of total distance (p>0.05) [73]
… total/relative distance:<br>Large to very large, significant correlations between total distance and session-RPE (r = 0.74 - 0.82; p<0.01) [59,60,93]<br>TD is among the strongest predictors of session-RPE using a predictive model [61]<br>… PL:<br>Large, significant correlations between PL and session-RPE (r = 0.76 - 0.84; p<0.01) [59,60]<br>Accumulated PL as a main contributor to RPE using least absolute shrinkage and selection operator (importance score: 0.487) [64]<br>Accumulated PL sideways as a main contributor to RPE using least absolute shrinkage and selection operator (importance score: 0.458) [64]<br>… acceleration/deceleration:<br>Small to moderate partial and within-individual correlations between RPE no. of accelerations/min (r = 0.25, 0.30, respectively) and between no. of accelerations and session-RPE (r = 0.37, 0.63, respectively) (p<0.001) [92]<br>Number of acceleration efforts between 2.0 - 3.5 m/s^2, > 3.5 m/s^2, decelerating distance between -3.5 - -2 m/s^2, and < −3.5 m/s^2 as main contributors to RPE using least absolute shrinkage and selection operator (importance score: 0.507, 0.515, 0.510, 0.466, respectively) [64]<br>… duration:<br>Total time is among the strongest predictors of session-RPE<br>Duration as a main contributor to RPE using least absolute shrinkage and selection operator (importance score: 0.471) [64]<br>… impacts:<br>Trivial to moderate adjusted correlations between number of impacts and RPE (r = 0.09 and session-RPE (r = 0.45) (p<0.001) [92]<br>… RHIE:<br>The average of RHIE per bout of 21s as a main contributor to RPE using least absolute shrinkage and selection operator (importance score: 0.513) [64]	Very likely to most likely moderate effects of HI running (>19.8 km/h) on CK 1, 24, 48, and 72h post-match using a linear mixed model (ES = 0.60-1.08) [86]<br>Significant correlation between number of sprints (>18 km/h) and sprint distance and change in CK (r = 0.78-0.80, p < 0.05) [70]<br>Very large correlations between sprint distance (>18 km/h) and HI distance (15–18 km/h) and % change in CK (r = 0.92-0.93, p<0.05) [70]<br>… total/relative distance:<br>Likely small to moderate effects of TD on CK 1, 24, and 72, but not 48h, post-match (ES = 0.33-0.66) [86]<br>… acceleration/deceleration:<br>+1 in CK Z-score corresponded to a moderate reduction of −4.3 ± 2.9% and 4.1 ± 2.9% in total accelerations (R^2 = 0.42; CL:0.09 ± 0.03), and total decelerations (R^2 = 0.42; Z-score coeff. ± 90% CL:0.09 ± 0.03) using mixed-effect linear models (p<0.05), compared to those without increased CK [75]<br>Likely small to moderate effects of HI events (sum of acceleration, deceleration, and change of direction events >2.5 m/s) on CK 1, 24, and 72, but not 48h, post-match (ES = 0.33-0.66) [86]<br>… duration:<br>Likely small to moderate effects of duration on CK 1, 24, and 72h post-match (ES = 0.33-0.66) (unclear effects 48h post-match) [86]<br>… maximal velocity:<br>+1 in CK Z-score corresponded to a moderate reduction of −4.6 ± 1.9% in maximal velocity (R^2 = 0.43; Z-score coeff. ± 90% CL:0.16 ± 0.08; p<0.05) using mixed-effect linear models, compared to those without increased CK [75]<br>… metabolic power:<br>+1 in CK Z-score corresponded to a moderate reduction of −2.1 ± 1.4% in high metabolic power distance[6] (R^2 = 0.65; Z-score coeff. ± 90% CL: 0.08 ± 0.03; p<0.05) using mixed-effect linear models, compared to those without increased CK [75]<br>… explosive distance:

[6] Indicator of HI distance: distance covered >25.5 W/kg; corresponds to running at constant speed of ~19.8 km/h on grass or accelerating or decelerating >2 m/s2.

… body load:

session-RPE was a significant, yet weak, predictor of total body load using linear regression analysis (β = 0.23, p < 0.05); the method accounted for 5% of the variance in total body load only [62]

… impulse load:

Very large correlations between impulse load and session-RPE (r = 0.84; p<0.001) [93]

… work:rest ratio[3]:

Small significant correlations between work:rest ratio and session-RPE (r = -0.29, p<0.01) [60]

Effindex[4]:

Mean Effindex of 1.3-1.4 during the matches with significant differences between playing positions (p<0.05) [81] and between halves [85]

EXERCISE-INDUCED RESPONSES

HR-based indices and …

… distance in speed zones:

Trivial relationship between morning HRV and HS distance (≥19.8km/h) (ES<0.2) [71]

Small significant correlations between HRV (expressed as Ln RMSSD) and fluctuations in total HI distance (>14.4km/h) (r = -0.24, p<0.05) [83]

Negligible and nonsignificant correlations between fluctuations in post-exercise HR recovery, HRV, and total HI distance (>14.4km/h) (p>0.05) [83,84]

… total/relative distance:

Trivial relationship between morning HRV and TD (ES <0.2) [71]

… acceleration/deceleration:

Trivial relationship between morning HRV and acceleration/deceleration (ES<0.2) [71]

… impacts:

Small relationship between morning HRV and impacts (ES=~0.3) [71]

… metabolic power:

+1 in CK Z-score corresponded to a moderate reduction of 3.1 ± 2.9% in explosive distance (R^2 = 0.59; Co-efficient ± 90% CL:0.15 ± 0.09; p<0.05) using mixed-effect linear models, compared to those without increased CK [75]

Myoglobin (as a muscle damage indicator) and …

… distance in speed zones:

Very likely moderate effects of HI running (>19.8 km/h) on myoglobin 1h post-match (ES = 0.8) (unclear effects 24, 48h post-match) [86]

No significant correlations between number of sprints (>18km/h)and change in myoglobin or % change in myoglobin [70]

… PL:

Likely small effects of TD on MYO 1h post-match (ES = 0.49) (unclear effects 24, 48h post-match) [86]

… total/relative distance:

Likely small effects of TD on MYO 1h post-match (ES = 0.58) (unclear effects 24, 48h post-match) [86]

… acceleration/deceleration:

Likely moderate effects of HI events (sum of acceleration, deceleration, and change of direction events >2.5 m/s) on MYO 1h post-match (ES = 0.68) (unclear effects 24, 48h post-match) [86]

…duration:

Likely moderate effects of duration on myoglobin 1h post-match (ES = 0.65) (unclear effects 24, 48h post-match) [86]

Body mass measures and …

… distance in speed zones:

No significant relationship between distances in speed zones and Δ in body mass during a match (p>0.05) [90]

… total/relative distance:

No significant relationship between TD and body mass changes during a match (p>0.05) [90]

C-reactive protein (CRP) and …

… distance in speed zones:

[3] distance covered at ≥ 4 km/h (work) divided by the distance covered at < 3.9 km/h (rest)

[4] Ratio of m/min/%HR_{max}

Small relationship between morning HRV and high metabolic load distance (ES=~0.4) [71]

… equivalent distance[5]:

Most likely a large relationship between morning HRV and equivalent distance index (ratio between equivalent distance and TD) (ES=1.89) [71]

Well-being questionnaire and …

… external load parameters:

External load indicators (duration, TD, PL, distance >20km/h, no. of acceleration and deceleration efforts) predicted fatigue, general muscle soreness and stress levels using gradient boosted regression trees with trivial effects sizes [68]

No significant correlations between accumulated weekly external load indicators (TD, PL 2D, distance >80% of maximum speed, acceleration >(-)2m/s^2) and total quality recovery questionnaire [87]

… distance in speed zones:

-1 in wellbeing Z-score corresponded to a -18±2m (-3.5±1.1%) reduction in total HSR (≥19.8–25.2 km/h) distance, and 4±1m (-4.9±2.1%) in sprint (≥25.2 km/h) distance using mixed-effect linear models [76]

Significant large correlations between fluctuations in fatigue and variability in previous-day HI distance (>14.4km/h) (r = -0.51, p<0.001) [83]

Negligible and nonsignificant correlations between variability in muscle soreness, sleep quality, and previous-day HI distance (>14.4km/h) (p>0.05) [83,84]

Small to moderate correlations between fluctuations in fatigue and HSR distance (>14.4km/h) covered on the previous 2, 3, and 4 days (r = -0.31, -0.42, and -0.28, respectively) (p<0 .05) [84]

Moderate correlation between HSR distance (18.0-21.6 km/h) and stress using the recovery-stress questionnaire (RESTQ) (r = 0.42) as well as between MI distance (7.21–13.32 km/h) and recovery (r = 0.33) (p<0.05) [88]

… total/relative distance:

Significant small correlations between TD and wellness score (r = -0.28, p<0.05) [63]

… PL:

Very large significant negative correlation between total HSR distance (>20 km/h) and CRP during one season (r = -0.863, p=0.027) [89]

ADAPTATION PARAMETERS

HR-based indices and …

… distance in speed zones:

Moderate correlations between total 10-week sprinting distance (>20.0 km/h) and % difference in HR_{max} after 10 weeks (r = 0.51, CI: -.37;.91, p<0.05) [82]

… duration:

Increase in ACWR-based session time significantly reduced HRV throughout a season (-7.4 +/- 3.6 ms; p<0.05) using mixed effects models [77]

Body mass measures and …

… distance in speed zones:

Large correlations between total 10-week sprinting distance (>20.0 km/h) and %Δ in body mass after 10 weeks (r = 0.70, CI: -.09;.95, p<0.05) [82]

… total/relative distance:

Large correlations between total 10-week TD and %Δ in body mass after 10 weeks (r = 0.53, CI: -.34; .91, p < 0.05) [82]

Increase in TD, but not relative distance, significantly predicted % body mass loss during training using step-wise linear regression analysis (r^2 = 0.253, p<0.001) [78]

… duration:

Duration was not a significant predictor of % body mass loss during training using step-wise linear regression analysis (p>0.05) [78]

… average speed:

Average speed was not a significant predictor of % body mass loss during training using step-wise linear regression analysis (p=0.59) [78]

… training load score by Polar:

Polar's training load score was not a significant predictor of % body mass loss during training using step-wise linear regression analysis (p=0.70) [78]

Strength test and …

… PL:

[5] accounts for distance covered and the intensity at which the distance was covered.

-1 in wellbeing Z-score corresponded to a 25±3AU/min (-4.9±3.19%) likely moderate reduction in PL/min [76]<br>… acceleration/deceleration:<br>Significant moderate correlations between deceleration efforts and wellness score (r = -0.27, p<0.05) [63]<br>… duration:<br>Significant moderate correlations between duration and wellness score (r = -0.35, p<0.05) [63]<br>… maximal velocity:<br>-1 in wellbeing Z-score corresponded to a 0.9±0.1km/h (-3.1±2.1%), 1±1 (-4.6±2.9%) likely and very likely moderate and large reduction in maximal velocity, and maximal velocity exposures, respectively [76]<br>… external/internal load ratios:<br>-1 in wellbeing Z-score corresponded to a -0.49±0.12m/min (trivially small), -1.20±0.08m/min (likely trivial), -0.02±0.01AU/min in TD:RPE (trivially small), total HSR distance(≥19.8–25.2 km/h):RPE and PL slow:RPE, respectively [76]<br>**CMJ and …**<br>… distance in speed zones:<br>-1 in CMJ Z-score pre-training corresponded to a −2.9 ± 0.5% and −3.5 ± 1.1% reduction in very HSR (>14.4 km/h) and HSR distance (>14.4 km/h), respectively (R^2 = 0.41; Z-score coeff. ± 90% CL: 0.07 ± 0.04; p<0.05) [75]<br>Moderate correlations between HI distance, HI distance/min, HSR distance, number of sprints/min and change in peak power output (calculated with CMJ parameters) at 24h (but not 48h) post-match (r = -0.349 - -0368; -p≤0.05) [74]<br>Small correlations between fluctuations in CMJ performance and total HI distance (>14.4 km/h) (r = 0.23, p<0.05) [83]<br>Negligible and nonsignificant correlations between fluctuations in CMJ performance and total HI distance (>14.4 km/h) (p>0.05) [84]<br>Trivial or unclear effects of HI distance (>19.8km/h) on CMJ height at 1, 24, 72h post-match, except for a likely small effect at 48h post-match (ES = 0.40) [86]<br>… total/relative distance:<br>Trivial or unclear effects of TD on CMJ height at 1, 24, 48, 72h post-match [86]	Large correlations between accumulated PL and % difference in peak torques at the hamstrings after 10 weeks (r = 0.62-0.68; CI: -0.22; 0.94) [82]<br>… distance in speed zones:<br>Very large correlations between total 10-week sprinting distance (>20.0 km/h) and quadriceps/hamstrings ratio (right & left: r= .84-.92, CI: .27 - .99, p<0.05) [82]<br>… acceleration/deceleration:<br>Large correlations between total 10-week acceleration and quadriceps left peak torque, and hamstring peak torque left & right (r = 0.62-.68, CI: -.22 - .94) after a 10-week period [82]<br>… duration:<br>Large inverse correlations between total 10-week training duration and %Δ in peak torque of left and right quadriceps and left hamstring (r = -0.56 - -0.7; CI: -.95 - .31, p<0.05) after a 10-week period [82]<br>… total/relative distance:<br>Large correlations between total 10-week TD and %Δ in left & right quadriceps/hamstring ratio after 10 weeks (r = 0.58 - 0.7, CI: -.28 - .95, p < 0.05) [82]<br>**VO_{2max} and …**<br>… PL:<br>Large correlations between accumulated PL and % difference in VO_{2max} after 10 weeks (r = 0.58, CI: -0.29; 0.92) [82]<br>… acceleration/deceleration:<br>Large correlations between total 10-week acceleration and %Δ of VO_{2max} after a 10-week period (r = 0.58, CI: -.29-.92, p<0.05) [82]<br>… duration:<br>Large inverse correlations between total 10-week training duration and %Δ in VO_{2max} (r = -0.58, CI: -.92-.31) after 10 weeks [82]<br>**30-15 Intermittent fitness test and …**<br>… distances in speed zones:<br>Unclear correlations between very HI running (>19.8km/h) and changes in the final velocity of the 30-15 intermittent fitness test after 5 weeks (r>0.05) [72]<br>… total/relative distance:<br>Unclear correlations between TD and changes in the final velocity of the 30-15 intermittent fitness test after 5 weeks (r = 0.33) [72]

… PL:<br>Trivial or unclear effects of PL on CMJ height at 1, 24, 48, 72h post-match [86]<br>… acceleration/deceleration:<br>-1 in CMJ Z-score pre-training corresponded to a moderate reduction of −5.3 ± 2.9% and 3.8 ± 2.9% in total accelerations and decelerations, respectively (R^2 = 0.39; Z-score coeff. ± 90% CL: 0.09 ± 0.03; p<0.05) [75]<br>Trivial or unclear effects of HI events on CMJ height at 1, 24, 48, 72h post-match [86]<br>Moderate correlation between acceleration >2m/s^2 during match in relation to accumulated weekly acceleration distance >2m/s^2 and absolute fatigue ($CMJ_{pre-match}$/CMJ_{max}) (r = -0.309, p<0.05) [87]<br>No significant correlations between total number of accelerations and decelerations and Δ in peak power output (calculated with CMJ parameters) at 24h and 48h post-match (p>0.05) [74]<br>Moderate correlations between number of HI accelerations and HI accelerations/min (but not HI decelerations) and Δ in peak power output (calculated with CMJ parameters) at 24h (but not 48h) post-match (r = -0.39; p<0.05) [74]<br>… duration:<br>Trivial or unclear effects of duration on CMJ height at 1, 24, 48, 72h post-match [86]<br>… metabolic power:<br>CMJ Z-score of -1 corresponded to a moderate reduction of −2.1 ± 1.4% in high metabolic power distance (R^2 = 0.45; Z-score coeff. ± 90% CL:0.08, ± 0.03; p<0.05) [75]<br>… maximal velocity:<br>CMJ Z-score of -1 corresponded to a moderate reduction of −5.6 ± 1.2% in maximal velocity (R^2 = 0.40; Z-score coeff. ± 90% CL:0.16 ± 0.08; p<0.05) [75]<br>… explosive distance:<br>CMJ Z-score of -1 corresponded to a moderate reduction of 1.1 ± 2.9% in explosive distance (R^2 = 0.41; Z-score coeff. ± 90% CL:0.15 ± 0.09; p<0.05) [75]<br>**Immunoglobulin and …**<br>… distance in speed zones:	… PL:<br>Large correlations between accumulated PL and changes in the final velocity of the 30-15 intermittent fitness test after 5 weeks (r = 0.54) [72]<br><br>INDIVIDUAL CHARACTERISTICS<br>**VO_{2max} and …**<br>distance in speed zones:<br>VO_{2max} among the strongest predictors of HI distance (>13km/h) during a game using stepwise regression (R = 0.989, SEE = 115.5 m, p = 0.001) [67]<br>Significant correlations between VO_{2max} and HI distance (>13km/h) (r = 0.755; p = 0.012) [67]<br>... total/relative distance:<br>Significant correlations between VO_{2max} and TD (r = 0.831; p<0.005) [67]<br>**Muscle Characteristics and …**<br>… distance in speed zones:<br>Leg thickness among the strongest predictors of HI distance (>13km/h) during a game (R = 0.989, SEE = 115.5 m, p<0.005) [67]<br>… distance in speed zones:<br>Dominant leg muscle pennation angle among the strongest predictors of HI distance (>13km/h) during a game (R = 0.989, SEE = 115.5 m, p<0.005) [67]<br>**RSA test and …**<br>… external load indicators (total/relative distance, distance in speed zones, PL, work rate):<br>No significant relationship across the season (p>0.05) [66]<br>**Sprint test and …**<br>… total/relative distance:<br>Likely small effects of TD on 30m sprint time 72h post-match (ES = 0.56) [86]<br>… PL:<br>Likely small effects of PL on 30m sprint time 72h post-match (ES = 0.46) [86]<br>**Yo-Yo intermittent recovery test (YYIR) and …**<br>… distance in speed zones:<br>In the late in-season and in early in-season, significant correlations between LI distance and YYIR2 performance (r = 0.88, 0.55, respectively, p<0.05) [66]

<table>
<tr>
<td colspan="4">Small negative associations between salivary secretory immunoglobulin A (sIgA) and HSR distance (>14.4 km/h) (r = -0.23), and number of sprints (>19.8 km/h) (r = -0.18) [91]
<u>… total/relative distance:</u>
Very likely a large negative correlations between sIgA and TD (r = -0.55) [91]
Trivial associations between sIgA and distance/min (r = 0.01) [91]
Negative significant relationship between TD and sIgA baseline change (r = −0.64- -0.69, p ≤ 0.05) [69]
<u>… acceleration/deceleration:</u>
Large negative correlations between sIgA and accelerations, and decelerations (r = -0.48 - -0.52) [91]
CK and …
<u>… distance in speed zones:</u>
+1 in CK Z-score corresponded to a −5.5 ± 1.1%, and −3.9 ± 0.5% reduction in total HSR (>14.4 km/h) and very HSR (>19.8 km/h) distance (R^2 = 0.31; Z-score coeff. ± 90% CL:0.07 ± 0.04) using mixed-effect linear models, compared to those without increased CK (p<0.05) [75]
Large correlation between % increase in CK concentration and number of sprints during a match (r = 0.88, p<0.05) [70]
Significant correlations between HI distance (r = 0.386), HI distance/min (r = 0.365), HSR distance (r = 0.363), number of sprints/min (r = 0.410) and Δ CK at 24h post-match (p<0.05). (No significant correlations after 48h of recovery) [74]</td>
<td colspan="2">In pre-season, significant correlations between MI distance and YYIR2 performance (r = 0.83, p<0.05) [66]
<u>… PL:</u>
Large correlations between PL and YYIR2 performance, in the late in-season (r = 0.67, p<0.05) [66]
<u>… total/relative distance:</u>
Moderate to large correlations between TD and YYIR2 performance, in the late in-season and in pre-season (r = 0.81, 0.65, respectively, p < 0.05) [66]
In the late in-season, in pre-season and in early in-season, moderate to large correlations between relative distance and YYIR2 performance (r = 0.77, 0.80, 0.58, respectively, p<0.05) [66]</td>
</tr>
<tr>
<td>Tag Football</td>
<td>[94]</td>
<td>Regional (n = 16, male)</td>
<td>Acceleration/Deceleration (m·s⁻²) (n = 1)
Distance in speed zones (m) (n = 1)
RHIE (n) (n = 1)
Total/relative distance (m, m/min) (n = 1)</td>
<td>INDIVIDUAL CHARACTERISTICS
CMJ (cm) (n = 1)
Sprint test (m/s) (n = 1)
YYIR (m) (n = 1)</td>
<td>INDIVIDUAL CHARACTERISTICS
CMJ and …
<u>... distance in speed zones:</u>
Moderate to large correlations between CMJ and very HSR m/min (≥18.1 km/h), very HSR efforts/min and peak running speeds (stronger correlations for inside than outside players) [94]
<u>… RHIE:</u>
Moderate to large correlations between CMJ performance and total RHIE for inside players (r = 0.46–0.85), weaker correlations for outside players (r = 0.01–0.49) [94]
Sprint test and …
<u>... distance in speed zones:</u></td>
</tr>
</table>

	Moderate to large correlations between straight line running speed (20m) and very HSR m/min (≥18.1 km/h) (r = 0.44–0.69), very HSR efforts/min (r = 0.44–0.50) and peak running speeds (r = 0.48–0.69) (stronger correlations for inside than outside players) [94]<br>… RHIE:<br>Moderate to very large correlations between straight line running speed and RHIE total bouts for inside players (r = 0.46–0.85); weaker correlations with outside players (r = 0.01–0.49) [94]<br>**YYIR and …**<br>… total/relative distance:<br>Positive correlation between YYIR2 and TD (r = 0.59–0.73), and relative distance (0.26–0.50) [94]<br>... distance in speed zones:<br>Positive correlation between YYIR2 and very HSR m/min (≥18.1 km/h) (r = 0.38–0.61), very HSR efforts/min (r = 0.55–0.58), and peak running speeds (r = 0.40–0.56) [94]<br>Moderate correlations between YYIR2 and RHIE total bouts (r = 0.48–0.52), and mean efforts per RHIE bout (r = 0.27 – 0.48) [94]

ACWR acute-to-chronic-workload-ratio, *ANN* artificial neural network analyses, *AU* arbitrary units, *CI* confidence interval, *CK* creatine kinase, *CL* confidence level, *CMJ* countermovement jump, *CRP* C-reactive protein, *ES* effect size, *GEE* Generalized estimating equations, *HI* high-intensity, HR_{max} maximal heart rate, *HR* heart rate, *HRV* heart rate variability, *HS* high-speed, *HSR* high-speed running, *LDH* Plasma lactate dehydrogenase, *LI* low intensity, *Ln RMSSD* natural logarithm of the square root of the mean of the sum of squares of differences between adjacent normal R-R intervals, *MAS* Maximal aerobic speed, *MI* moderate intensity, *MSR* moderate-speed running, *NT-proBNP* Urinary N-terminal prohormone of brain natriuretic peptide, *PA* pennation angle, *PL* PlayerLoad, *RHIE* repeated high-intensity events, *RPE* rating of perceived exertion, *RSA* repeated sprint ability, *sIgA* salivary secretory immunoglobulin A, T_{core} core temperature, *TD* total distance, *TRIMP* training impulse, VO_2max maximal oxygen uptake, *VT* ventilatory threshold, *YYIR* Yo-Yo intermittent recovery test, χ^2 Wald chi-square
